# Exome sequencing identifies mutations in *ABCD1* and *DACH2* in two brothers with a distinct phenotype

**DOI:** 10.1186/s12881-014-0105-6

**Published:** 2014-09-19

**Authors:** Yanliang Zhang, Yanhui Liu, Ya Li, Yong Duan, Keyun Zhang, Junwang Wang, Yong Dai

**Affiliations:** Department of Clinical Laboratory, the First Affiliated Hospital of Kunming Medical University, Yunnan Province, 650032 P R China; Prepotency Center, Dongguan Maternal and Child Health Hospital, Guangdong Province, 523000 P R China; Radiology Department, Dongguan Guanghua Hospital of Tongji Medical College, Guangdong Province, 523000 P R China; The Clinical Medical Research Center, Second Clinical Medical College (Shenzhen People’s Hospital), Jinan University, Guangdong Province, 518020 P R China

**Keywords:** ABCD1, Adrenoleukodystrophy, DACH2, Distinct phenotype, Exome sequencing

## Abstract

**Background:**

We report on two brothers with a distinct syndromic phenotype and explore the potential pathogenic cause.

**Methods:**

Cytogenetic tests and exome sequencing were performed on the two brothers and their parents. Variants detected by exome sequencing were validated by Sanger sequencing.

**Results:**

The main phenotype of the two brothers included congenital language disorder, growth retardation, intellectual disability, difficulty in standing and walking, and urinary and fecal incontinence. To the best of our knowledge, no similar phenotype has been reported previously. No abnormalities were detected by G-banding chromosome analysis or array comparative genomic hybridization. However, exome sequencing revealed novel mutations in the *ATP-binding cassette, sub-family D member 1* (*ABCD1*) and *Dachshund homolog 2* (*DACH2*) genes in both brothers. The *ABCD1* mutation was a missense mutation c.1126G > C in exon 3 leading to a p.E376Q substitution. The *DACH2* mutation was also a missense mutation c.1069A > T in exon 6, leading to a p.S357C substitution. The mother was an asymptomatic heterozygous carrier. Plasma levels of very-long-chain fatty acids were increased in both brothers, suggesting a diagnosis of adrenoleukodystrophy (ALD); however, their phenotype was not compatible with any reported forms of ALD. *DACH2* plays an important role in the regulation of brain and limb development, suggesting that this mutation may be involved in the phenotype of the two brothers**.**

**Conclusion:**

The distinct phenotype demonstrated by these two brothers might represent a new form of ALD or a new syndrome. The combination of mutations in *ABCD1* and *DACH2* provides a plausible mechanism for this phenotype.

## Background

Adrenoleukodystrophy (ALD; OMIM#300100) is a serious progressive, genetic disorder that affects the adrenal glands, the spinal cord and the white matter of the nervous system. It is thought to be caused by genetic defects in the *ATP-binding cassette, sub-family D member 1* (*ABCD1*) (OMIM*300371) gene [1,2]. ALD is characterized by variations in phenotypic expression; seven ALD forms have been reported so far, including childhood cerebral, adolescent cerebral, adult cerebral, adrenomyeloneuropathy (AMN), Addison-only, asymptomatic or presymptomatic, and olivo-ponto-cerebellar ALD [3,4]. However, there is no exact genotype-phenotype correlation between *ABCD1* defects and clinical phenotype. Furthermore, the combination of defects in *ABCD1* with defects in other genes, such as the hemophilia A gene [5] or *DXS1357E* [6], can complicate and worsen the clinical phenotype.

We present two brothers with a distinct phenotype including congenital language disorder, growth retardation, severe intellectual disability, inattention, dysphoria, drooling, heterophony, difficulty in walking and standing without aid, standing on tiptoe with aid, urinary incontinence, and fecal incontinence. To the best of our knowledge, no cases with a similar phenotype have been reported previously. We performed cytogenetic tests and exome sequencing in the two brothers and their parents to screen for potential pathogenic causes.

## Methods

### Subjects

Two brothers and their parents, as well as 500 unaffected individuals (250 males and 250 females) of matched geographical ancestry, were enrolled in this study. The study was approved by the Shenzhen People’s Hospital Ethics Committee, which abides by the Helsinki Declaration on ethical principles for medical research involving human subjects. Written informed consent was obtained from all the participants or their guardians.

### DNA extraction

Genomic DNA was obtained from peripheral blood lymphocytes from all individuals, using standard procedures [7].

### Cytogenetic analysis

G-banding chromosome analysis (~850 bands) was performed on cultures of peripheral blood lymphocytes from the two brothers and their parents, according to standard techniques [7].

### Array comparative genomic hybridization

Array comparative genomic hybridization (array-CGH) was performed using Agilent Technologies’ Array CGH Kits (Santa Clara, CA, USA). This platform is 60-mer oligonucleotide-based microarray that allows genome-wide survey and molecular profiling of genomic aberrations with a resolution of ~20 kb (Kit 244A). DNAs were labeled by random priming (Agilent Technologies) for 2 h using Cy5-dUTP for test DNAs and Cy3-dUTP for reference DNAs. Labeled products were column-purified. After probe denaturation and pre-annealing with 50 μg of Cot-1 DNA, hybridization was performed at 65°C with rotation for 40 h. After two washing steps, the arrays were analyzed using an Agilent scanner and Feature Extraction 10.5.0.1 software. The data were analyzed using CGH Analytics 4.0 software (Agilent Technologies). The Aberration Detection Method 2 algorithm was used to identify aberrant intervals.

### Exon capture and sequencing, read mapping and single nucleotide polymorphism detection

Targeted capture and massive parallel sequencing of approximately 201,904 coding exons from genomic DNA from the two patients and their mother were performed using the Agilent SureSelect Human All Exon kit, following the manufacturer’s protocols. Briefly, genomic DNA was sheared by sonication and the DNA fragments were then purified using a QIAquick PCR Purification Kit (Qiagen, Hilden, Germany). The fragment ends were repaired and adaptors were ligated to the fragments (NEBNext DNA sample prep, New England Biolabs). The adapter-ligated templates were purified using Agencourt AMPure SPRI beads and fragments with an insert size of about 250 bp were excised. Extracted DNA was amplified by ligation-mediated polymerase chain reaction, purified, and hybridized to the SureSelect Biotinylated RNA Library ‘baits’ (Agilent) for enrichment. Each captured library was then loaded on a HiSeq 2000 platform for sequencing. Raw image files were processed using Illumina Pipeline (v1.6) for base-calling with default parameters. SOAPaligner (v2.01) was used to align the sequencing reads to the NCBI human genome reference assembly (build 36.3). Reads that aligned to the designed target region were collected for single nucleotide polymorphism (SNP) identification and subsequent analysis. The consensus sequence and quality of each allele was calculated by SOAPsnp.

### Detection of insertions and deletions

Insertions and deletions (Indels) in the exome regions were identified by *de novo* assembly of the sequencing reads. The reads were assembled using SOAPdenovo with the 31-mer option enabled and the assembled consensus sequences were then aligned to the reference genome by LASTZ. The alignment result was passed to axtBest to separate orthologous from paralogous alignments. Finally, breakpoints in the alignment were identified and the genotypes of Indels were annotated.

### Variant analysis

To distinguish between potentially pathogenic mutations and other variants, we only focused on non-synonymous (NS) variants, splice acceptor and donor site mutations (SS), and short coding Indels, anticipating that synonymous variants would less likely to be pathogenic. The variants were compared and filtered using public databases, including dbSNP (v129), 1000 Genome Project (20100208 release), eight HapMap exomes, and YH genome. A novel variant was defined as one that did not exist in these datasets. Only recessive models of inheritance (autosomal recessive model and X-linked recessive model) were considered because of the normal phenotypes of the parents.

### Mutation validation

Variants detected by exome sequencing were validated by Sanger sequencing.

## Results

### Clinical findings

**Figure 1:**
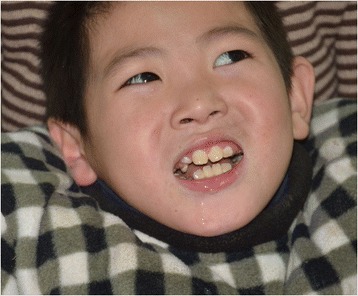
**The poorly developed permanent teeth of the patient 1.**

**Figure 2:**
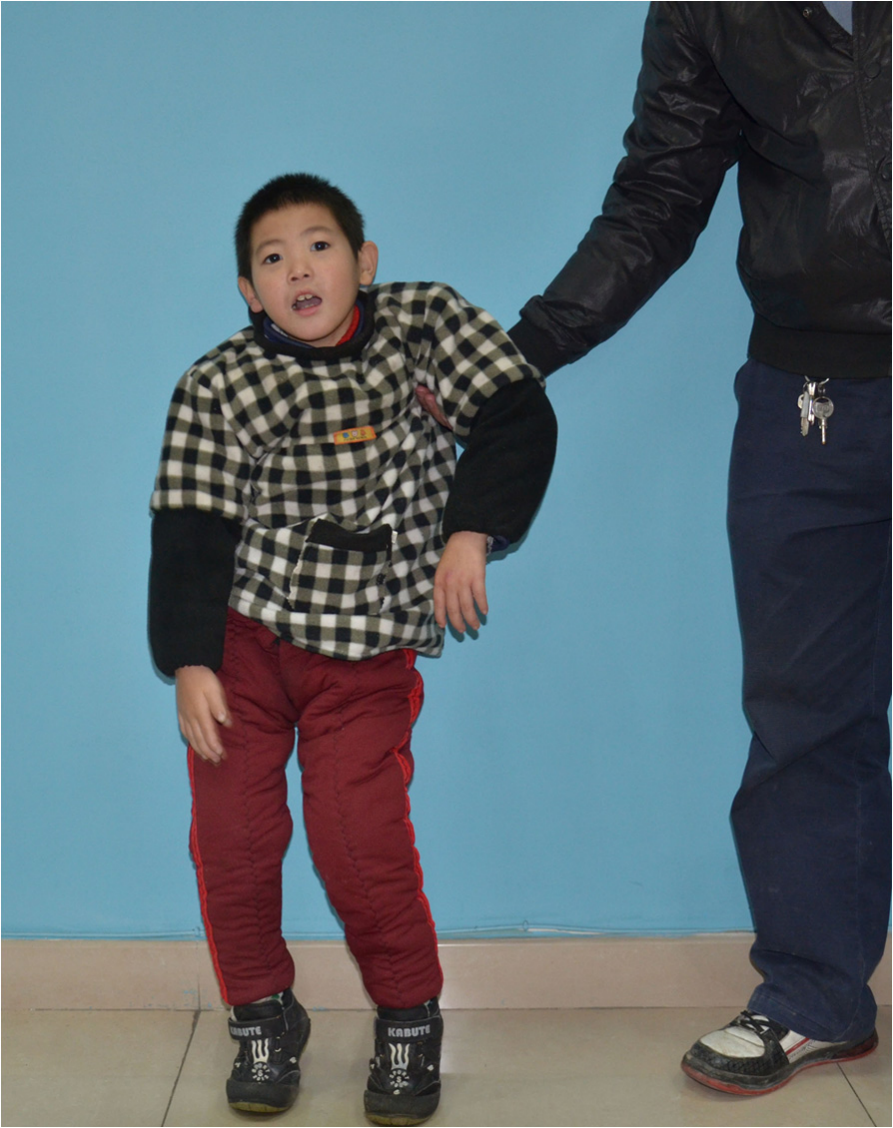
**Only with the aid, the patient 1 was able to stand on tiptoe.**

**Figure 3:**
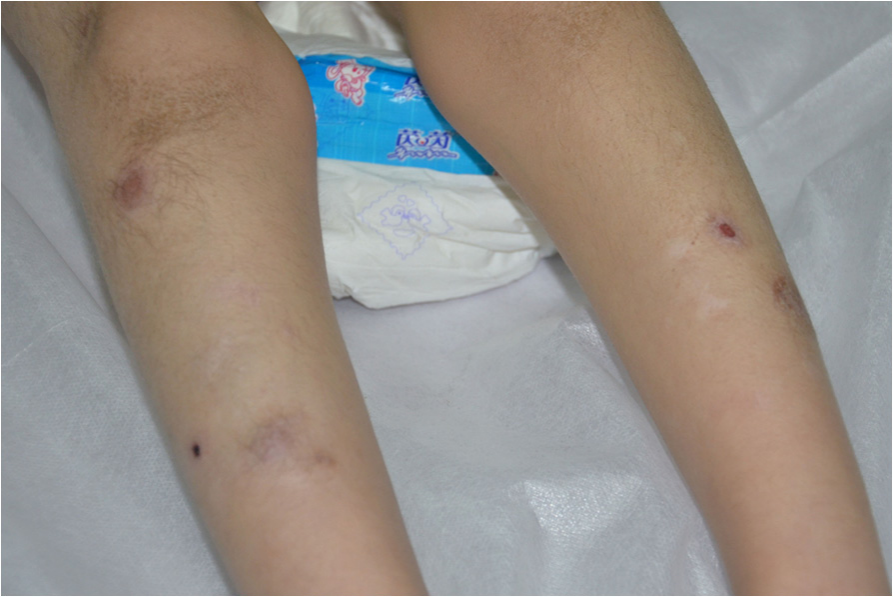
**The lower limbs of the patient 1 showing many wounds caused by frostbite or burn unconsciously.**

**Figure 4:**
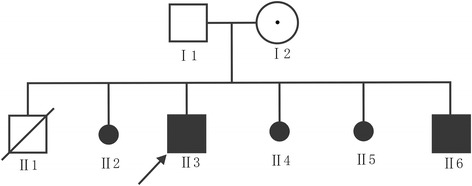
**The Pedigree of the family.** The open square: normal male individual; the closed square: affected male individual; the closed square with an arrow: male proband; the open square with a slash: deceased male individual; the small closed circle: abortion; the open large circle with a closed circle in center: asymptomatic female carrier.

Patient 1: A 10-year-old boy was the result of the third pregnancy of a 33-year-old, gravida six, para three, Chinese woman. The father was 34 years old. The parents were non-consanguineous and healthy. The pregnancy was uncomplicated and the mother denied any exposure to alcohol, teratogenic agents, irradiation or infectious diseases during the pregnancy. The boy was born at full-term and delivered by spontaneous vaginal delivery. His birthweight was 2,500 g (3rd–10th centile), length was 46 cm (<3rd centile), and head circumference was 30 cm (<3rd centile). His growth continued to be retarded, and his weight was 28.5 kg (<50th centile), length was 101 cm (<3rd centile), and head circumference was 46.5 cm (<3rd centile) at 8 years old. He was noted to have global developmental delay, particularly affecting expressive language. He had never spoken using phrases or ‘word combinations’. His hearing and vision were normal. He had severe intellectual disability (IQ < 25) and response retardation. His cognition, attention and memory were impaired. He was often restless, crying and drooling, was interested in strangers or novel things, and was frequently incontinent. He had dental dysplasia, with aberrant, dust-colored teeth; the maxillary teeth 11 and 21 were reversed, the 21 and 22 dental crowns were incompletely erupted, bilateral deciduous canines were ablated, and the distal ends of both maxillary and mandibular permanent dental crowns were moderately or mildly tilted (Figure 1). His motor skills were impaired: his manual dexterity and finger tapping were poor, he was able to pick up chopsticks or a spoon, but could not pick up small items or thread a needle. He was never able to walk or stand without aid, and with aid, was only able to achieve a wobbling walk on tiptoe (Figure 2). He was unable to raise or flex his lower limbs freely, and they were ice-cold and insensitive to pain, heat and cold, leading to frequent frostbite and burning (Figure 3). Laboratory tests, including white blood cell and platelets counts, bleeding time, sedimentation rate, serum electrolytes and protein, blood glucose, immune electrophoresis, C-reactive protein, C3 complement, autoantibody screening, and antistreptolysin titer revealed no pathologic findings. The concentrations of cortisol and adrenocorticotropic hormone (ACTH) were normal, though most sex hormones were lower than the reference values (Table 1). The plasma level of very-long-chain fatty acids (VLCFA) was elevated, with a C24/C22 ratio of 1.342 (reference interval: 0.788–1.090), and C26/C22 ratio of 0.086 (reference interval: 0.018–0.038). Sonography, X-ray examination, magnetic resonance imaging (MRI) and computed tomography (CT) scan showed no obvious abnormalities. The mother had previously aborted three times and her first child died of liver cancer at the age of 6 years. There was no family history of chromosomal anomaly or similar case, until the birth of a younger brother (patient 2) (Figure 4).

**Table 1:** **The hormones detection results of two brothers**

**Tests**	**Results**		**Reference intervals**
	**Patient 1**	**Patient 2**	
Triiodothyronine (T3)	1.79	2.35	1.3 ~ 3.1 nmol/L
Thyroxine (T4)	112.9	121.6	66 ~ 181 nmol/L
Free triiodothyronine (FT3)	4.13	5.28	3.10 ~ 6.80 pmol/L
Free thyroxine (FT4)	16.34	16.18	12 ~ 22 pmol/L
Thyrotropic-stimulating hormone (TSH)	3.30	3.70	0.27 ~ 4.20 uIU/ml
Luteinizing hormone (LH)	0.549	0.234	1.70 ~ 8.60 mIU/ml
Follicle-stimulating hormone (FSH)	1.67	0.935	1.5 ~ 12.4 mIU/ml
Prolactin (PRL)	84.69	81.83	98 ~ 456 uIU/ml
Estradiol (E2)	24.05	18.35	49.2 ~ 218 pmol/L
Testosterone (T)	0.069	0.069	9.90 ~ 27.80 nmol/L
Progesterone (P)	0.095	0.095	0.70 ~ 4.30 nmol/L
Corticosteroid (CRO)	196.2	166.5	171 ~ 536 nmol/L
Adrenocorticotropic hormone (ACTH)	9.2	7.1	2.2 ~ 16.6 pmol/L

**Figure 5:**
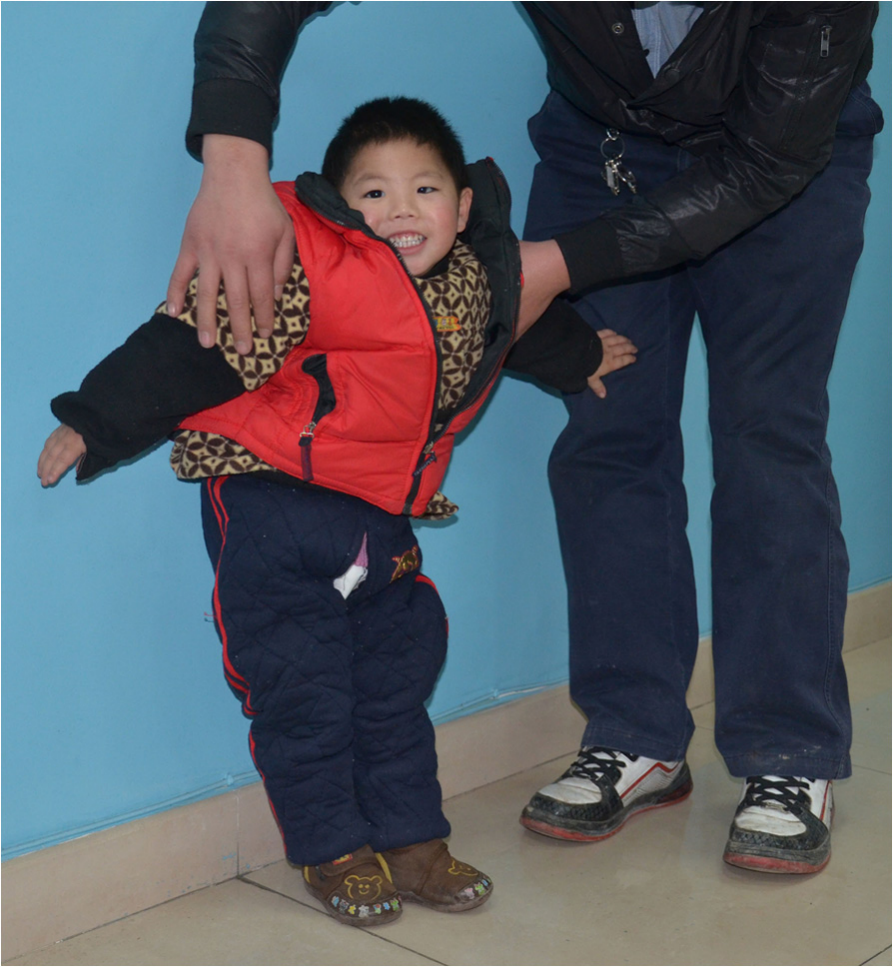
**The patient 2 was only able to stand with assistance.**

Patient 2: The younger brother of patient 1 was born as the sixth child, 6 years after patient 1, at 40 weeks of gestation after an uncomplicated pregnancy and delivery. His birthweight was 2500 g (3rd–10th centile), length was 45 cm (<3rd centile), and head circumference was 31 cm (3rd–10th centile). He started learning to walk at 1 year old, but his lower limbs were weak. He failed to thrive, and his weight was 9 kg (<3rd centile), length was 80.5 cm (<3rd centile), and head circumference was 42.5 cm (<3rd centile) at 2.5 years. He had severe intellectual disability (IQ < 25) and response retardation. He was inattentive and often drooling, restless and crying. The muscular tension in his lower limbs was high, and his muscle strength was low. He was unable to stand, walk or speak, and was only able to stand with assistance at the age of 3 years (Figure 5). He was frequently incontinent. Sonography, X-ray examination, MRI and CT scan showed no obvious abnormalities. Auditory brainstem response examination revealed a high frequency hearing threshold in both ears (left ear: 60 db, right ear: 105 db). The concentration of ACTH was normal, but cortisol and sex hormones were low (Table 1). The plasma level of VLCFA showed an increased C24/C22 ratio of 1.428 (reference interval: 0.788–1.090), and C26/C22 ratio of 0.092 (reference interval: 0.018–0.038). No obvious abnormalities were found by other laboratory tests.

### Genetic findings

The karyotypes of the two brothers and their parents were normal according to G-banding chromosome analysis. No submicroscopic chromosome aberrations were detected by array-CGH.

The exomes in the two brothers and their mother were sequenced. A total of 35,495,497 bases in 244,069 exons from 21,326 genes were targeted for sequencing. An average of 3.8 Gb of effective sequence was generated per individual as 90 bp paired-end reads; 2.9 Gb (76.3%) passed the quality assessment and aligned to the human reference sequence, and 63.3% of the total bases mapped to the targeted bases with a mean coverage of 64.3-fold (Table 2). About 96% of targeted bases were sufficiently covered to pass our threshold for variant calling. After identification of variants, we focused on NS, SS and Indels, which were more likely to be pathogenic mutations than other variants. Each sibling had one or more NS/SS/Indel variants in ~3,000 genes. Using public databases (dbSNP129, 1000 Genomes Project, eight HapMap exomes and YH genome) as filters, we identified 51 and 33 novel mutated genes in the two brothers, respectively. We compared these genes from the two brothers with their mother to exclude variants that were not compatible with an autosomal recessive model or X-linked recessive model. This reduced the number of candidate genes to 14 and eight, respectively. Three candidate genes were shared by both brothers: one autosomal gene *GNAQ* (OMIM*600998) and two X chromosomal genes *ABCD1* and *DACH2* (OMIM*300608) (Table 3).

**Table 2:** **Summary of original exome sequencing data**

**Sample**	**Bases (Gb)**	**Map bases (Gb)**	**Map bases rate (%)**	**Exon map bases (Gb)**	**Exon map bases rate (%)**	**Exon length (Mb)**	**Covered length (Mb)**	**Coverage (%)**	**Mean sequencing depth**
Patient 1	3.93	3.04	77.35	2.50	63.61	37.81	37.47	99.10	66.01
Patient 2	3.52	2.67	75.85	2.22	63.07	37.81	37.41	99.00	58.70
mother	4.05	3.13	77.28	2.57	63.46	37.81	37.35	99.00	68.22

**Table 3:** **Identification of the candidate genes for two brothers by exome sequencing**

**Filter**	**Case 1**	**Case 2**
NS/SS/I	2983	2981
Not in dbSNP129, 1000 Genome Project, eight HapMap exomes, and YH genome	51	33
Comparing to the mother	14	8
Shared by two cases	3	

Each cell indicates the number of genes with nonsynonymous (NS) variants, splice acceptor and donor site mutations (SS) and coding indels (I). Rows show the effect of excluding from consideration variants found in dbSNP129, 1000 Genome Project, the eight HapMap exomes, and the YH genome. Columns show the effect of requiring that NS/SS/Indel variants be observed in each case.

**Figure 6:**
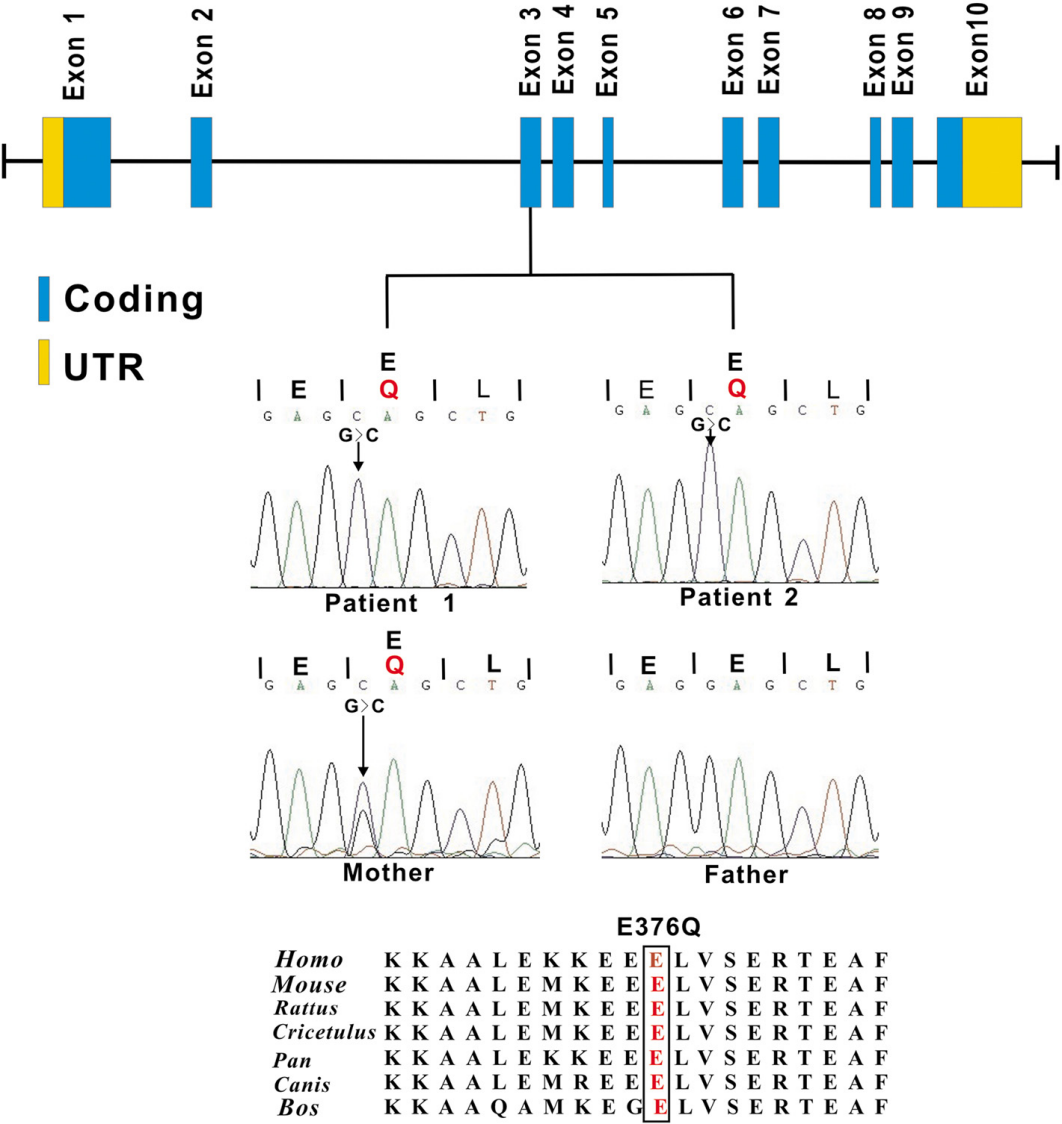
**Genomic structure of the exons encoding the open reading frame of** ***ABCD1*** **and identified mutations.** *ABCD1* is composed of ten exons that encode untranslated regions (UTR) (orange) and protein coding sequence (blue) (upper panel). Sanger sequence of codons 375–377 in exon 3 of *ABCD1* indicated the same mutation was present in two brothers and their mother (middle panel). E376Q missense mutation was at a highly conserved position in *ABCD1* shown by comparison to the corresponding sequence of six vertebrates (lower panel). Rattus = Rattus norvegicus; Cricetulus = Cricetulus griseus; Pan = Pan troglodytes; Canis = Canis lupus familiaris; Bos = Bos Taurus.

**Figure 7:**
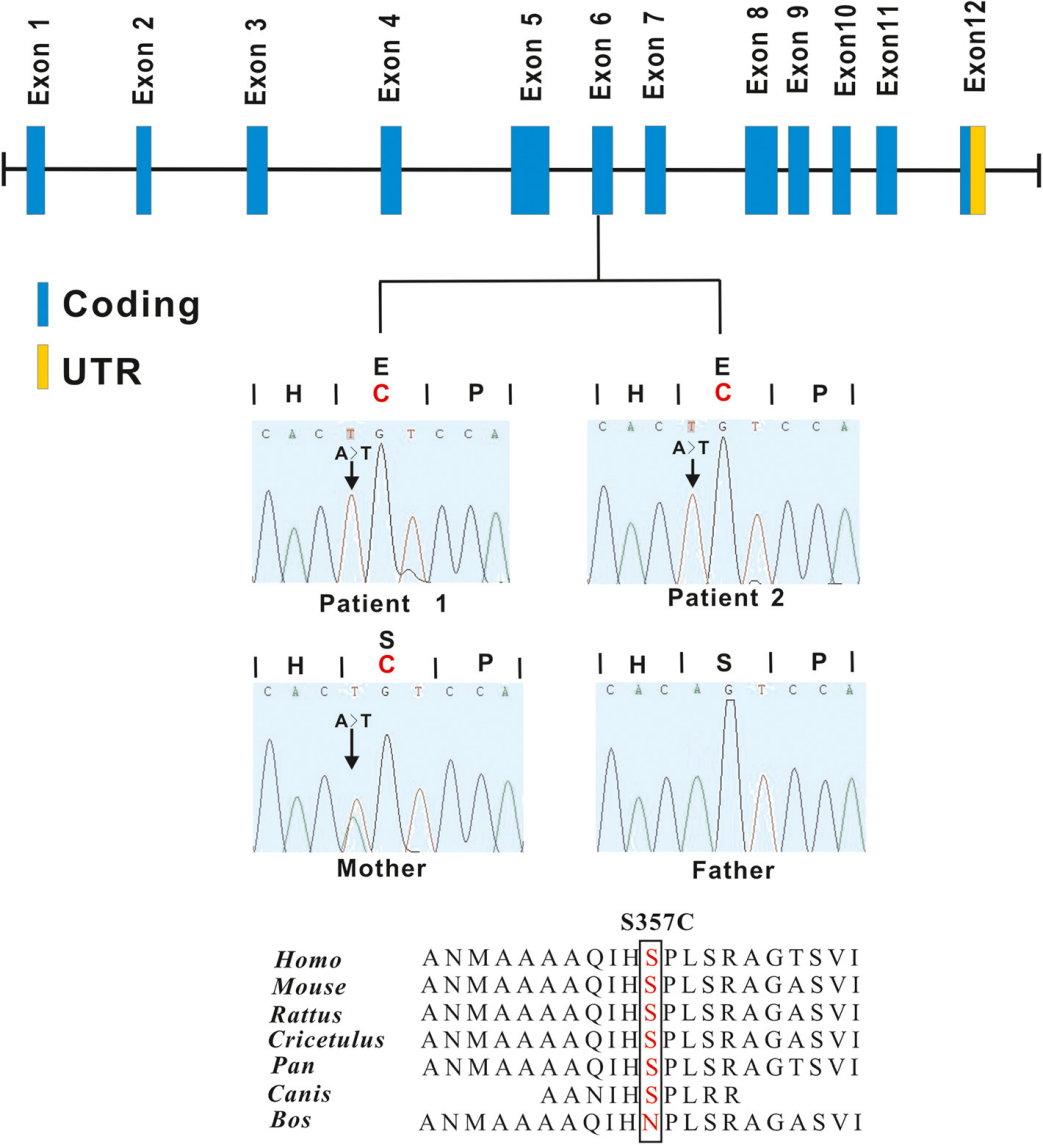
**Genomic structure of the exons encoding the open reading frame of** ***DACH2*** **and identified mutations.** *DACH2* is composed of twelve exons that encode untranslated regions (UTR) (orange) and protein coding sequence (blue) (upper panel). Sanger sequence of codons 356–358 in exon 6 of *DACH2* indicated the same mutation was present in two brothers and their mother (middle panel). S357C missense mutation was at a highly conserved position in *DACH2* shown by comparison to the corresponding sequence of six vertebrates (lower panel). Rattus = Rattus norvegicus; Cricetulus = Cricetulus griseus; Pan = Pan troglodytes; Canis = Canis lupus familiaris; Bos = Bos Taurus.

Sanger sequencing demonstrated that the *GNAQ* mutations were false positives, but the mutations in *DACH2* and *ABCD1* were accurate. The *ABCD1* mutation was a missense mutation c.1126G > C in exon 3, leading to a p.E376Q substitution (Figure 6), and the *DACH2* mutation was also a missense mutation c.1069A > Tc.1069A > T in exon 6, leading to ap.S357C substitution (Figure 7). The mother was an asymptomatic heterozygous carrier (Table 4). Sanger sequencing of 500 unaffected individuals of matched geographical ancestry failed to detect the *ABCD1* mutation, while the *DACH2* mutation was present in one female. BLAST analysis (http://blast.ncbi.nlm.nih.gov/Blast.cgi) indicated that both the mutations, c.1126G > C transition (E376Q) of *ABCD1* and c.1069A > T transition (p.S357C) of *DACH2*, occurred in highly conserved positions (Figure 6, 7). To assess the likelihood that the two variants had functional impacts on the respective proteins, the biophysical consequences of these variants were predicted using PolyPhen-2. The variant c.1126G > C transition (E376Q) of *ABCD1* was considered likely to be functionally benign, while the variant c.1069A > T transition (p.S357C) of *DACH2* was likely to be functionally damaging.

**Table 4:** **The exome sequencing and sanger sequencing results of the 3 candidate genes**

**Gene**	**Chromosome**	**Position**	**Reference**	**Exome sequencing**			**Sanger sequencing**			
		**(NCBI36/hg18)**		**Patient 1**	**Patient 2**	**Mother**	**Patient 1**	**Patient 2**	**Mother**	**Father**
*GNAQ*	9	79,726,915	G	G/T	G/T	G	G	G	G	G
*GNAQ*	9	79,726,932	T	T/A	T/A	T/A	T	T	T	T
*DACH2*	X	85,856,344	A	T	T	A/T	T	T	A/T	A
*ABCD1*	X	152,654,804	G	C	C	C/G	C	C	C/G	G

## Discussion

We report on two brothers who presented with a distinct phenotype. The potential pathogenic cause was investigated using G-banding chromosome analysis and high-resolution array-CGH in the two brothers and their parents, but no abnormalities were detected. Exome sequencing was therefore performed on the brothers and their mother, to detect genetic variations. The small sample and uncertain pathogenesis of the disease meant that both autosomal recessive and X-linked recessive models were possible. The rarity of the disorder makes it unlikely that causative variants would be present in the general population, and we therefore compared our detected variants with those in dbSNP129, 1000 Genomes Project, eight HapMap exomes and YH genome to eliminate shared variants. Sequencing and comparison of the coding region from the affected brothers and their unaffected mother, and filtering out of the benign changes using public databases, led to the identification of three candidate genes: one autosomal gene *GNAQ* and two X chromosomal genes *ABCD1* and *DACH2*. Subsequent Sanger sequencing showed that the mutations in *GNAQ* were false positives.

The *ABCD1* mutation was a novel missense mutation, c.1126G > C transition (E376Q), in exon 3. Several lines of evidence support a causative role for this mutation in the brothers’ phenotype: 1) *ABCD1* mutation is consistent with an X-linked recessive model, which was one of the two possible inherited models; 2) the *ABCD1* mutation is not present in the public databases, including dbSNP129, 1000 Genomes Project, eight HapMap exomes and YH genome, and was not found in 500 normal ethnicity-matched controls, excluding the possibility of an amino acid substitution polymorphism; and 3) comparative analyses of *ABCD1* in other species show that E376 is conserved among primates, rodents, and other vertebrate species.

*ABCD1* encodes an integral peroxisomal membrane protein (ALD protein) that belongs to the ATP-binding cassette–transporter superfamily [6]. The peroxisomal ATP-binding cassette transporter is involved in the import of VLCFA into the peroxisome. Defects in *ABCD1* have been shown to be associated with impaired peroxisomal β-oxidation and accumulation of saturated VLCFA in all tissues of the body, and are considered to be the underlying cause of ALD [1,3].

ALD is a rare X-linked demyelinating disorder affecting the nervous system, adrenal cortex and testis [1,2], and is characterized by variation in phenotypic expression [6,8,9]. To date, several clinical forms have been reported in male patients (Table 5) [3,4,10-12]. The cerebral forms, including childhood cerebral, adolescent cerebral and adult cerebral, are associated with an inflammatory reaction in the cerebral white matter and progressive neurological damage with rapid evolution, leading to a vegetative state within 6 months to 2 years after onset of symptoms, followed by death at variable ages [13,14]. In contrast, AMN mainly involves the spinal cord and peripheral nerves, the inflammatory response is absent or mild, and its progression is slower. AMN usually presents with initial symptoms in adult men aged from 20 years to middle-age. Affected individuals develop progressive stiffness and weakness in the legs, sphincter control abnormalities, and sexual dysfunction, and may also have serious cognitive and behavioral disturbances over the decades [12]. Addison-only individuals present with signs of adrenal insufficiency between 2 years of age and adulthood; the signs include unexplained vomiting, weakness or coma. Individuals with asymptomatic ALD usually have a biochemical abnormality, but no manifestation of adrenal or neurologic disease. The cerebellum and brain stem are usually involved in individuals with olivo-ponto-cerebellar ALD, who present with signs between adolescence and adulthood.

**Table 5:** **Phenotypic comparison between reported male ALD forms and two brothers**

**Forms**	**Description**	**Two brothers**
Childhood cerebral	Onset at 3–10 years of age with a peak at seven years. This form virtually never occurs before three years of age. Affected boys present with progressive behavioural and cognitive neurological deficits, such as inattention, hyperactivity, deterioration in handwriting skills, diminishing school performance; difficulty in understanding speech, spatial orientation; clumsiness; visual disturbances; and aggressive behavior. Brain MRI examination can be strikingly abnormal even when symptoms are relatively mild. Most individuals have impaired adrenocortical function at the time that neurological disturbances are first noted. Total disability often within 3 years.	Onset before 2 years of age. Presentation is not progressive, including congenital language disorder, intellectual disability, growth retardation, response retardation, dysphoria, drooling; difficulty in walking and standing; urinary incontinence and fecal incontinence; movement and sensory dysfunctions of lower limbs; normal brain MRI, normal adrenocortical function.
Adrenomyeloneuropathy (AMN)	Onset at 28 ± 9 years, progressive stiffness and weakness of legs, abnormalities of sphincter control, sexual dysfunction, distal axonopathy, inflammation mild or absent, mainly spinal cord involvement, cerebral involvement later in 45% of cases.	
Adolescent cerebral	Like childhood cerebral, but onset at 10–21 years of age. Somewhat slower progression.	
Adult cerebral	Dementia, behavioral disturbances. Rapid inflammatory cerebral progression resembling the childhood form, without preceding AMN, onset after 21 years of age.	
Addison-only	Primary adrenocortical insufficiency without neurological abnormalities, including unexplained vomiting, weakness, coma, onset before 7.5 years of age.	
Asymptomatic or presymptomatic	ALD gene abnormality without neurological or endocrine abnormalities, further studies can reveal subclinical adrenal insufficiency or mild AMN phenotype. This form is common in boys under 4 years of age.	
Olivo-ponto-cerebellar	Cerebral and brain stem involvement, onset between adolescence to adulthood.	

The unique phenotype of the two brothers initially suggested a novel syndrome, rather than ALD. However, a missense mutation in *ABCD1* was unexpectedly identified in both brothers and their mother by exome sequencing. Although PolyPhen-2 predicted that the biophysical consequences of the variant c.1126G > C (E376Q) of *ABCD1* were likely to be functionally benign, other variants in the same exon, such as c.1114A > T (p.K372*) and c.1137C > G (p.S379R), have been reported to lead to ALD [15]. Further laboratory tests showed elevated plasma VLCFA levels in the brothers, and no mutations were detected in genes associated with other peroxisomal diseases, such as Zellweger syndrome, acyl-CoA oxidase deficiency, D-bi-functional protein deficiency, and b-ketothiolase deficiency. The brothers should be therefore diagnosed with ALD.

Nevertheless, the phenotype exhibited by the two brothers was not consistent with any reported ALD forms. Their ages at onset would suggest childhood cerebral ALD (CCALD), but their conditions were not progressive, their brain MRI results were normal, and they had severe congenital language and motor disorders and intellectual disability. In contrast, cognitive and motor development are normal in CCALD prior to the onset of demyelinating lesions visible at brain MRI, suggesting that the brothers’ phenotype differed from CCALD. In addition, both brothers had urinary and fecal incontinence, their ages at onset were younger than 2 years old, they had severe congenital intellectual disabilities, and were unable to walk and speak, indicating a phenotype incompatible with AMN and other known forms of ALD. The distinct phenotype displayed by the two brothers suggests that other pathogenetic factors, in addition to *ABCD1* mutation, may have been responsible for their condition.

*DACH2* is a homolog of *dachshund*. The *dachshund/Dach* gene family encodes transcriptional cofactors that are conserved between insects and vertebrate. *Drosophila dachshund* is a critical regulator of eye, brain, and limb formation, and null mutations in *dachshund* result in abnormal retinal, brain, genital, and limb development [16-19]. The vertebrate homologs *Dach1* and *Dach2* also play an important role in the development of the retina, brain and limbs [20]. *DACH2* encodes a transcription factor characterized by the presence of three conserved domains [21], which is involved in the regulation of organogenesis, myogenesis, brain and limb development [20,22]. The first domain (DD1) at the N-terminus (amino acids 66–162) and the second domain (DD2) at the C-terminus (amino acids 452–543) are highly conserved in all members of the DACH protein family and appear to be involved in DNA binding and in the interaction with EYA proteins, respectively [23,24]. A third domain DD3 is present in the central portion of the protein (amino acids 314–412) and is shared by all members of the DACH1 and DACH2 subfamilies, but its detailed function remains unknown. The variant c.1069A > T transition (p.S357C) of *DACH2* detected in the present study was located in the DD3 domain and was predicted to be functionally damaging by PolyPhen-2, suggesting a potential role in two brothers’ phenotype. The combined mutations in *ABCD1* and *DACH2* thus provide a plausible explanation for the abnormal phenotype observed in both brothers. However, further functional studies are needed to clarify the effects of these variants.

## Conclusion

The distinct phenotype demonstrated by two brothers might represent a new form of ALD or a new syndrome. The combination of mutations in *ABCD1* and *DACH2* provides a plausible mechanism for this phenotype.
